# Supplementary material for: A listening advantage for native speech is reflected by attention-related activity in auditory cortex
Source: Commun Biol. 2025 Feb 5;8:180. doi: 10.1038/s42003-025-07601-2 (PMC11799217; doi:10.1038/s42003-025-07601-2)
Supplement: Supplementary file 1 — Supplementary information [file 42003_2025_7601_MOESM1_ESM.pdf]

Supplementary Information

A listening advantage for native speech is reflected by attention-related activity in auditory cortex

Meng Liang, Johannes Gerwien, and Alexander Gutschalk

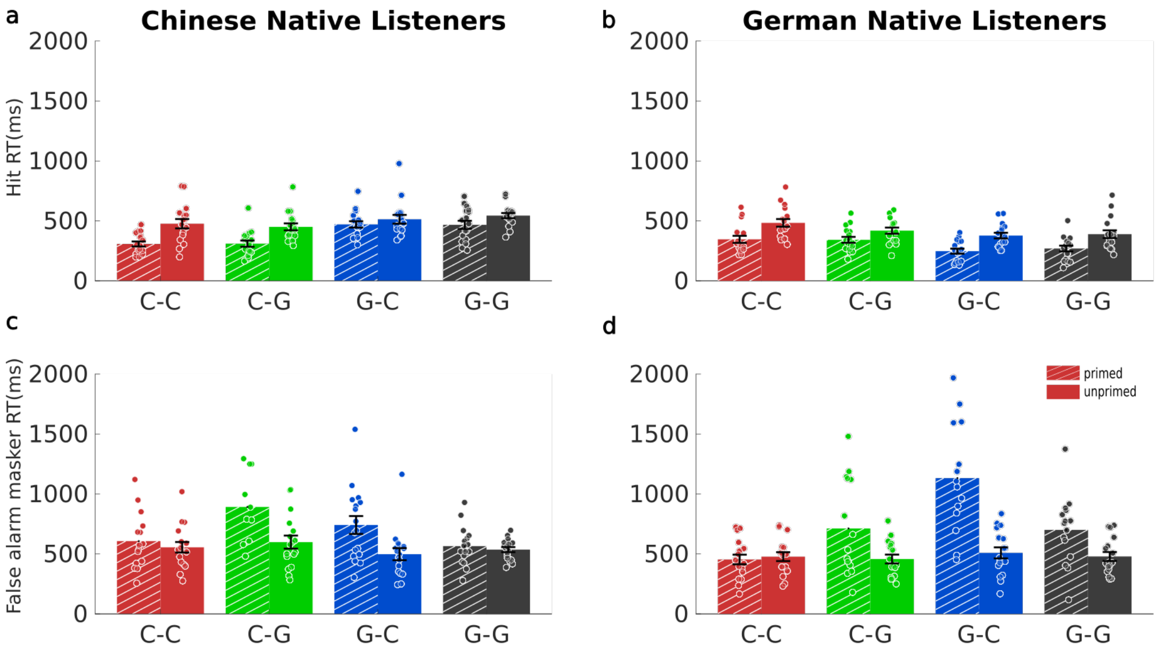

Supplementary Fig. 1 | Response time of button presses indicating detection of the target word. The response time for hit trials is shown in **a** for Chinese and in **b** for German native listeners (mean ± standard error; n=17 per group; circles indicate single participant data). The response time for false alarm trials is shown in **c** Chinese and in **d** for German native listeners. Stimulus language combinations are coded by color (red/C-C: consistent, Chinese target; black/G-G: consistent, German target; green/C-G: inconsistent, Chinese target; blue/G-C: inconsistent German target). Primed conditions are indicated by hatched, unprimed conditions by solid bars.

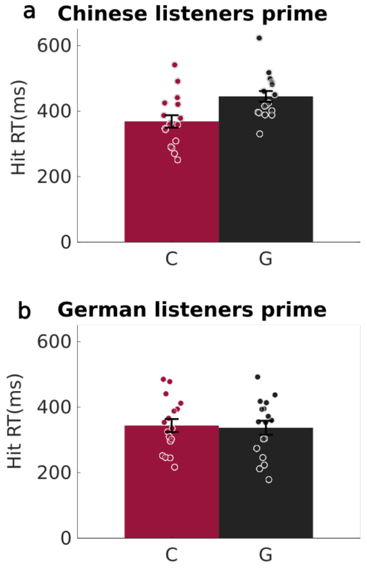

Supplementary Fig. 2 | Response time of button presses indicating detection of the target word for the prime. The response time for hit trials is shown in **a** for Chinese and in **b** for German native listeners (mean ± standard error; n=17 per group; circles indicate single participant data. Language effect: $F(1,32) = 8.767$, $p = 0.006$; language *group $F(1,32) = 12.530$, $p = 0.001$).

Supplementary Table 1. Statistical analysis of reaction time for hits.

Unprimed, two streams

Test	F (1,32)	p	partial η^2
Target language	0.005	0.944	0.000
Target language*group	13.25	<0.001	0.293
T-M consistency	4.715	0.037	0.128
T-M consistency*group	0.104	0.749	0.003
Target language*T-M consistency	0.663	0.422	0.02
Target language*T-M consistency*group	0.906	0.348	0.028

Primed, two streams

Test	F (1,32)	p	partial η^2
Target language	5.877	0.021	0.155
Target language*group	63.854	<0.001	0.666
T-M consistency	0.159	0.693	0.005
T-M consistency*group	0.406	0.529	0.013
Target language*T-M consistency	0.119	0.732	0.004
Target language*T-M consistency*group	0.148	0.703	0.005

Group (Native German listeners, n=17; native Chinese listeners, n=17); ANOVA for repeated measures with the factors Target language (German, Chinese) and target-masker (T-M) consistency (target and masker consistent or inconsistent language; two-stream conditions only).

Supplementary Table 2. Average dipole position and orientation (ori) in approximated Talairach coordinates (mean +/- standard deviation).

Chinese listeners

	X	Y	Z	X ori	Y ori	Z ori
left	-49.7 ± 6.8	-17.9 ± 9.2	5.7 ± 6.2	0.07 ± 0.13	0.42 ± 0.27	0.82 ± 0.24
right	54.5 ± 5.2	-11.2 ± 10.5	5.7 ± 9.5	-0.11 ± 0.13	0.30 ± 0.32	0.84 ± 0.28

German listeners

	X	Y	Z	X ori	Y ori	Z ori
left	-48.4 ± 7.3	-20.0 ± 8.5	7.4 ± 10.2	0.09 ± 0.16	0.39 ± 0.26	0.86 ± 0.11
right	47.1 ± 7.8	-12.9 ± 8.05	8.0 ± 10.3	-0.15 ± 0.17	0.30 ± 0.26	0.89 ± 0.09

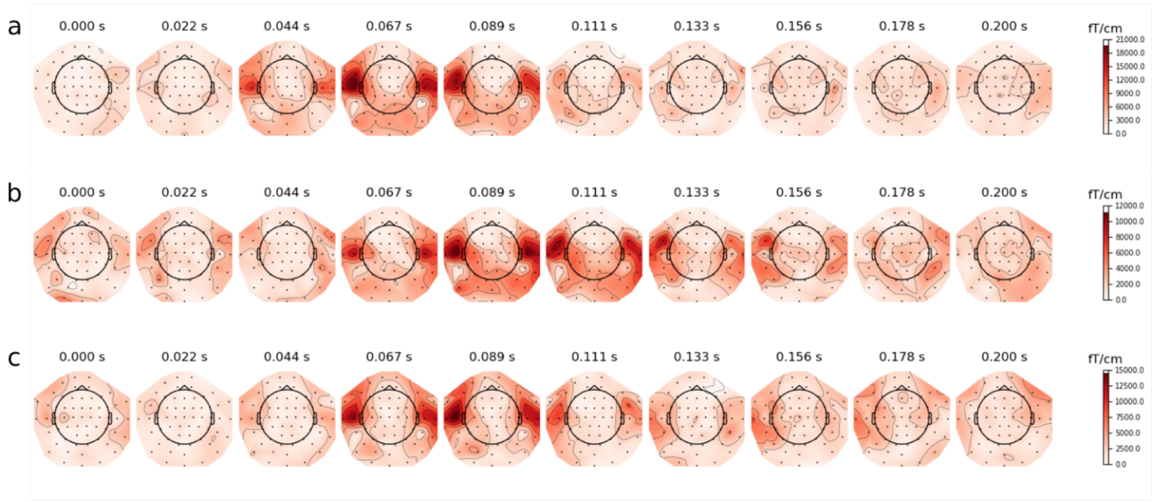

Supplementary Fig. 3 | Topographic map of grand average TRF response (n=34). Topographic maps are shown in 22-ms steps from 0 ms to 200 ms. Maps represent the absolute values of combined activity for each of the 61 planar gradiometer positions. **a** Single-stream prime TRFs. **b** Target stream TRFs of the speech mixture (two-stream conditions). **c** Masker stream TRFs of the speech mixture (two-stream conditions). As can be seen across all three conditions, the strongest activity is generally observed in gradiometers over both temporal lobes, matching to sources in the bilateral auditory cortex.

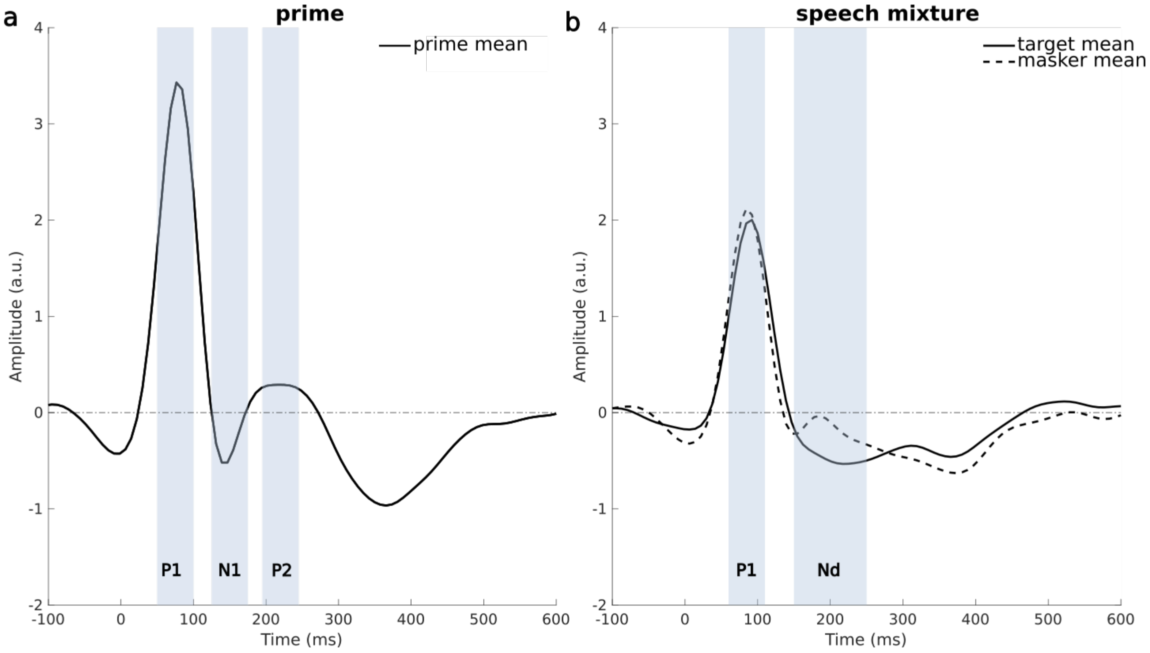

Supplementary Fig. 4 | Definition of analysis time intervals based on the grand-average temporal response function (TRF) in auditory cortex (n=34). Both source waveforms are based on the dipoles fitted to the P1 in the prime condition. **a** Mean TRF for the prime (single stream) conditions averaged across participants, languages, and hemispheres. The 50-ms-long analysis time windows (gray shading) were chosen to include the peak and avoid overlap with adjacent components, resulting in the time windows for P1: 50-100 ms, N1: 125-175 ms, and P2: 195-245 ms. **b** Mean TRF for the speech mixture (two-stream) conditions, averaged across all target (solid) and masker streams (dashed) across participants, languages, and hemispheres. The 50-ms-long time window for the P1 was centered around the peak (60-110 ms). The 100-ms-long time window for the Nd was defined after P1 offset and to include the first negative-going peak of the subsequent sustained response (N150-250 ms). Comparison of target and masker TRF shows that this time interval captures the grand-average Nd.

Supplementary Table 3. Statistical analysis of two-stream TRF source waveforms in the Nd time window

Complete data set, unprimed and primed

test	F (1,32)	p	partial η^2
attention	7.450	0.010	0.189
attention*group	0.617	0.438	0.019
stimulus language	1.176	0.286	0.035
stimulus language*group	7.122	0.012	0.182
T-M consistency	2.486	0.125	0.072
T-M consistency*group	1.548	0.222	0.046
prime	9.434	0.004	0.228
prime*group	0.820	0.372	0.025
attention*stimulus language	0.715	0.404	0.022
attention*stimulus language*group	13.159	<.001	0.291
attention*T-M consistency	8.351	0.007	0.207
attention*T-M consistency*group	0.000	0.999	0.000
stimulus language*T-M consistency	7.432	0.010	0.188
stimulus language*T-M consistency*group	2.677	0.112	0.077
attention*prime	0.030	0.864	0.000
attention*prime*group	0.315	0.578	0.010
stimulus language*prime	0.006	0.940	0.000
stimulus language*prime*group	0.000	0.985	0.000
T-M consistency*prime	3.422	0.074	0.097
T-M consistency*prime*group	0.159	0.692	0.005
attention*stimulus language*T-M consistency	0.075	0.785	0.002
attention*stimulus language*T-M consistency*group	0.764	0.389	0.023
attention*stimulus language*prime	3.263	0.080	0.093
attention*stimulus language*prime*group	0.291	0.594	0.009
attention*T-M consistency*prime	1.178	0.286	0.036
attention*T-M consistency*prime*group	0.377	0.544	0.012
stimulus language*T-M consistency*prime	0.090	0.766	0.003
stimulus language*T-M consistency*prime*group	0.812	0.374	0.025
attention*stimulus language*T-M consistency*prime	4.046	0.053	0.112
attention*stimulus language*T-M consistency*prime*group	2.668	0.112	0.077

Supplement Table 4. Statistical analysis of the envelope encoding accuracy for two-stream conditions.

test	F (1,32)	p	partial η^2
attention	8.564	0.006	0.211
attention*group	3.820	0.059	0.107
stimulus language	32.785	<.001	0.506
stimulus language*group	0.167	0.685	0.005
T-M consistency	1.312	0.261	0.039
T-M consistency*group	0.211	0.649	0.007
prime	5.694	0.023	0.151
prime*group	6.242	0.018	0.163
attention*stimulus language	0.013	0.909	0.000
attention*stimulus language*group	0.073	0.789	0.002
attention*T-M consistency	0.650	0.426	0.020
attention*T-M consistency*group	0.574	0.454	0.018
stimulus language*T-M consistency	5.686	0.023	0.151
stimulus language*T-M consistency*group	0.518	0.477	0.016
attention*prime	0.015	0.903	0.000
attention*prime*group	0.135	0.715	0.004
stimulus language*prime	0.159	0.693	0.005
stimulus language*prime*group	0.004	0.951	0.000
T-M consistency*prime	2.631	0.115	0.076
T-M consistency*prime*group	0.143	0.707	0.004
attention*stimulus language*T-M consistency	2.509	0.123	0.073
attention*stimulus language*T-M consistency*group	0.819	0.372	0.025
attention*stimulus language*prime	3.508	0.070	0.099
attention*stimulus language*prime*group	2.227	0.145	0.065
attention*T-M consistency*prime	1.535	0.224	0.046
attention*T-M consistency*prime*group	3.337	0.077	0.094
stimulus language*T-M consistency*prime	0.143	0.708	0.004
stimulus language*T-M consistency*prime*group	1.404	0.245	0.042
attention*stimulus language*T-M consistency*prime	0.491	0.489	0.015
attention*stimulus language*T-M consistency*prime*group	0.011	0.917	0.000

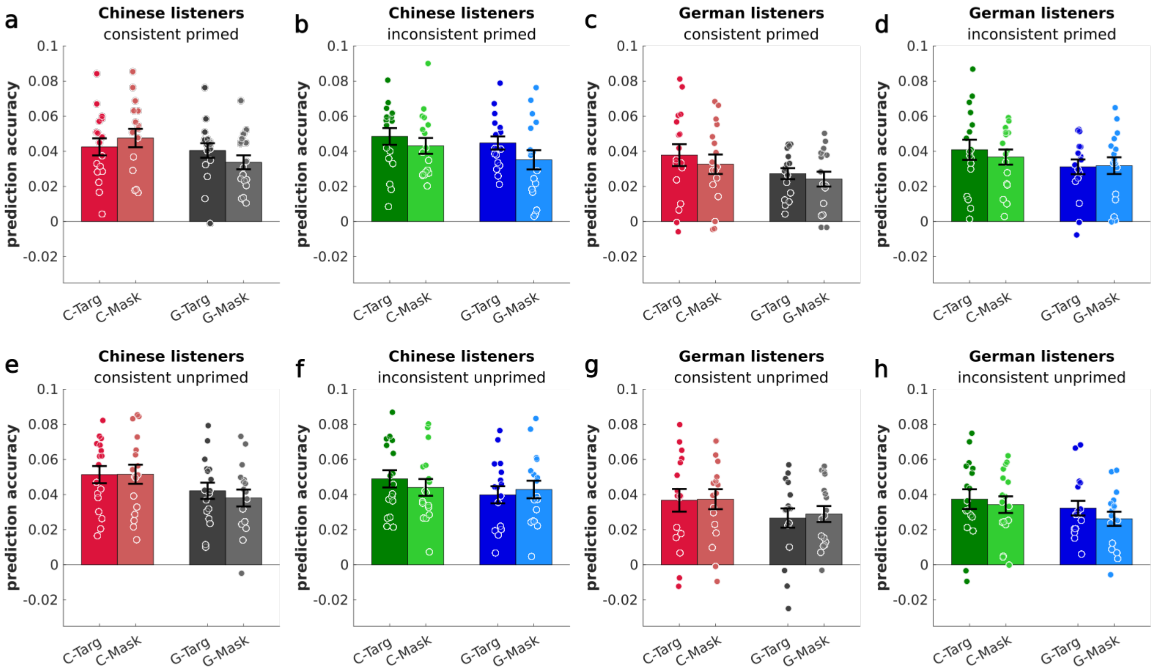

Supplementary Fig. 5 | Neural encoding of MEG source activity in auditory cortex in the P1 time window (60-110 ms). The measure indicates how well the continuous MEG activity can be estimated based on the envelope when convoluted with the TRF estimated in another data set. Each single-participant data (small circles) is based on an average of five data segments evaluated in a leave-one-out cross validation (bars indicate mean±standard error across participants; n=17 per group). In each pair of bars, the left (darker color) represents the target and the right (brighter color) the masker of the same language and subcondition. **a** Encoding accuracy for Chinese listeners for primed, consistent conditions. **b** Encoding accuracy for Chinese listeners for primed, inconsistent conditions. **c** Encoding accuracy for German listeners for primed, consistent conditions. **d** Encoding accuracy for German listeners for primed, inconsistent conditions. **e** Encoding accuracy for Chinese listeners for unprimed, consistent conditions. **f** Encoding accuracy for Chinese listeners for unprimed, inconsistent conditions. **g** Encoding accuracy for German listeners for unprimed, consistent conditions. **h** Encoding accuracy for German listeners for unprimed, inconsistent conditions.

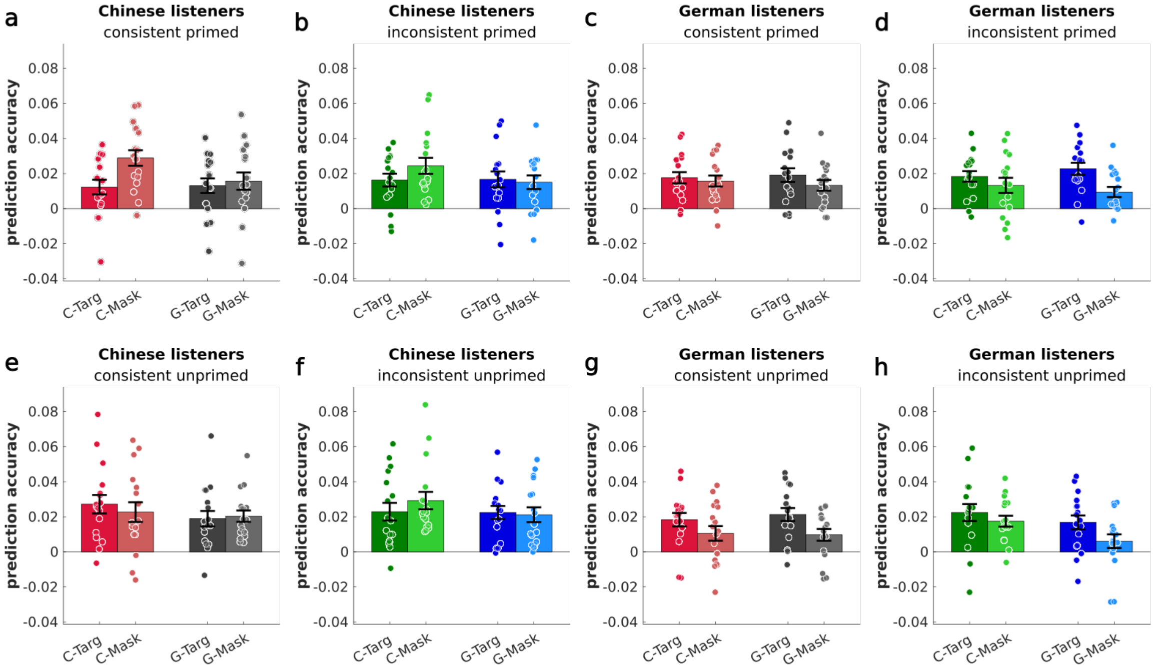

Supplementary Fig. 6| Neural encoding of MEG source activity in auditory cortex in the Nd time window (150-250 ms). Except for the time-interval used, same analysis and arrangement as in Fig. 5 and in Supplementary Fig. 4, see there for details.

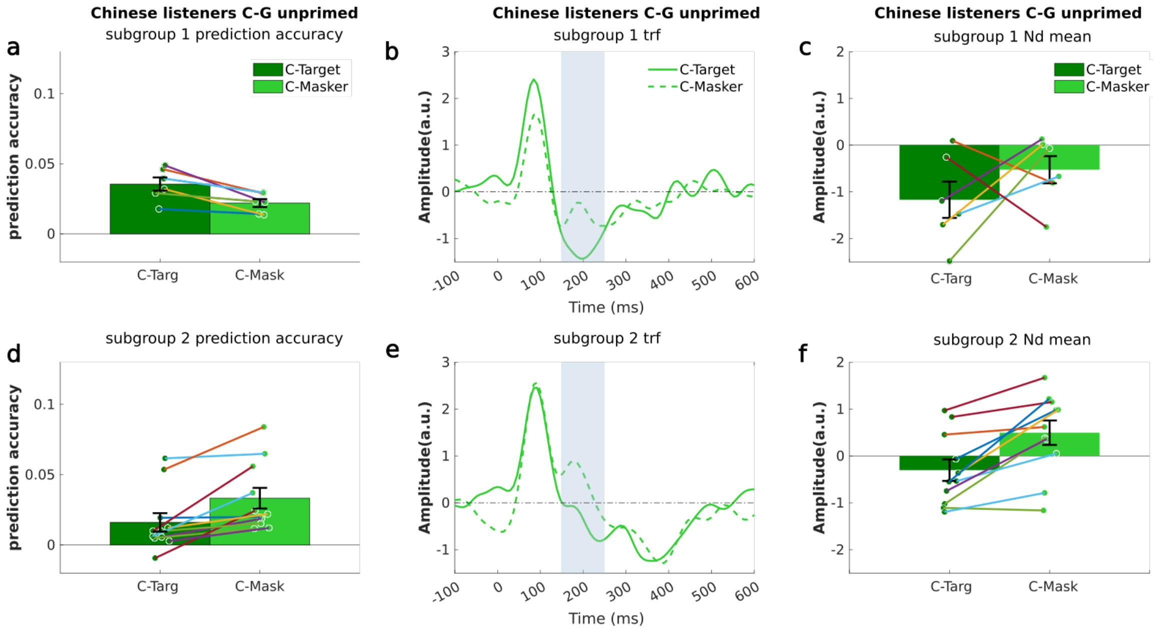

Supplementary Fig. 7 | Chinese-German unprimed condition for Chinese listeners separated into two subgroups. This condition was chosen, because it revealed opposite results between the TRF and encoding analysis in the same time window. The selection of subgroups (subgroup 1: a-c, n=7; subgroup 2: d-f, n=10) was based on the encoding accuracy in the Nd time window (cf. Supplementary Fig. 4). **a** All participants who showed higher encoding accuracy for target compared to masker streams were sorted into subgroup 1. **b** TRF source waveforms for subgroup 1, the Nd time window shaded in gray. **c** Amplitude in the Nd time window for target and masker. **d** All participants who showed lower encoding accuracy for target compared to masker streams were sorted into subgroup 2. **e** TRF source waveforms for subgroup 1, Nd time window shaded in gray. **f** Amplitude in the Nd time window for target and masker. Note that, while the encoding in subgroup 2 is stronger, the difference of target minus masker trials shows a similar, negative-going value, i.e. the Nd. However, the relative contribution of P2 is stronger in subgroup 2, and more so for masker streams, explaining the higher encoding accuracy for masker trials in subgroup 2. All panels indicate mean ± standard error; single-subject data indicated by connected circles.
