# Supplementary material for: A listening advantage for native speech is reflected by attention-related activity in auditory cortex
Source: Commun Biol. 2025 Feb 5;8:180. doi: 10.1038/s42003-025-07601-2 (PMC11799217; doi:10.1038/s42003-025-07601-2)
Supplement: Supplementary file 2 — Reporting Summary [file 42003_2025_7601_MOESM2_ESM.pdf]

nature portfolio

Corresponding author(s): Alexander Gutschalk

Last updated by author(s): Dec 11, 2024

Reporting Summary

Nature Portfolio wishes to improve the reproducibility of the work that we publish. This form provides structure for consistency and transparency in reporting. For further information on Nature Portfolio policies, see our Editorial Policies and the Editorial Policy Checklist.

Statistics

For all statistical analyses, confirm that the following items are present in the figure legend, table legend, main text, or Methods section.

n/a	Confirmed	
☐	☒	The exact sample size (*n*) for each experimental group/condition, given as a discrete number and unit of measurement
☐	☒	A statement on whether measurements were taken from distinct samples or whether the same sample was measured repeatedly
☐	☒	The statistical test(s) used AND whether they are one- or two-sided<br>*Only common tests should be described solely by name; describe more complex techniques in the Methods section.*
☐	☒	A description of all covariates tested
☐	☒	A description of any assumptions or corrections, such as tests of normality and adjustment for multiple comparisons
☐	☒	A full description of the statistical parameters including central tendency (e.g. means) or other basic estimates (e.g. regression coefficient) AND variation (e.g. standard deviation) or associated estimates of uncertainty (e.g. confidence intervals)
☐	☒	For null hypothesis testing, the test statistic (e.g. *F*, *t*, *r*) with confidence intervals, effect sizes, degrees of freedom and *P* value noted<br>*Give P values as exact values whenever suitable.*
☒	☐	For Bayesian analysis, information on the choice of priors and Markov chain Monte Carlo settings
☒	☐	For hierarchical and complex designs, identification of the appropriate level for tests and full reporting of outcomes
☒	☐	Estimates of effect sizes (e.g. Cohen's *d*, Pearson's *r*), indicating how they were calculated

Our web collection on statistics for biologists contains articles on many of the points above.

Software and code

Policy information about availability of computer code

Data collection	MEGIN/Neuromag recording software
Data analysis	mTRF toolbox, BESA 6.1

For manuscripts utilizing custom algorithms or software that are central to the research but not yet described in published literature, software must be made available to editors and reviewers. We strongly encourage code deposition in a community repository (e.g. GitHub). See the Nature Portfolio guidelines for submitting code & software for further information.

Data

Policy information about availability of data

All manuscripts must include a data availability statement. This statement should provide the following information, where applicable:

- Accession codes, unique identifiers, or web links for publicly available datasets
- A description of any restrictions on data availability
- For clinical datasets or third party data, please ensure that the statement adheres to our policy

The behavioral and MEG data will be made available at heiDATA, the online data repository of Heidelberg University. The data have now been uploaded to a repository including the regressors required for their evaluation under a private URL: https://heidata.uni-heidelberg.de/privateurl.xhtml?token=0ad0078f-3e32-470d-8b2b-a357b5eb6df3

Human research participants

Policy information about studies involving human research participants and Sex and Gender in Research.

Reporting on sex and gender	Self reported gender is reported but played no role for the analysis.
Population characteristics	17 Chinese native speakers between the age of 24 and 34 (10 female) who had little knowledge of and little exposure to German and 17 native speakers of German between the age of 19 and 33 (10 female) with no prior knowledge of Chinese were recruited. 13 of the Chinese participants had participated in a one-month German course before moving to Germany, but none of them reported to understand German when they participated in the experiment. The average time that Chinese participants had spent in Germany was 8.5 months (3 - 15months). All participants reported normal hearing.
Recruitment	Participants were recruited via campus adds.
Ethics oversight	Ethics committee of Heidelberg University Medical School.

Note that full information on the approval of the study protocol must also be provided in the manuscript.

Field-specific reporting

Please select the one below that is the best fit for your research. If you are not sure, read the appropriate sections before making your selection.

☐ Life sciences ☒ Behavioural & social sciences ☐ Ecological, evolutionary & environmental sciences

For a reference copy of the document with all sections, see nature.com/documents/nr-reporting-summary-flat.pdf

Behavioural & social sciences study design

All studies must disclose on these points even when the disclosure is negative.

Study description	behavioral and magnetoencephalography study
Research sample	2 groups with each 17 adult participants
Sampling strategy	based on language background (native German versus native Chinese speakers).
Data collection	participants performed a target-detection task while the magnetoencephalogram was recorded
Timing	recording time approximatly 1h
Data exclusions	none
Non-participation	no dropouts
Randomization	no randomization, groups were assigned based on language background

Reporting for specific materials, systems and methods

We require information from authors about some types of materials, experimental systems and methods used in many studies. Here, indicate whether each material, system or method listed is relevant to your study. If you are not sure if a list item applies to your research, read the appropriate section before selecting a response.

Materials & experimental systems

n/a	Involved in the study	
☒	☐	Antibodies
☒	☐	Eukaryotic cell lines
☒	☐	Palaeontology and archaeology
☒	☐	Animals and other organisms
☒	☐	Clinical data
☒	☐	Dual use research of concern

Methods

n/a	Involved in the study	
☒	☐	ChIP-seq
☒	☐	Flow cytometry
☒	☐	MRI-based neuroimaging
